# A pragmatic randomised controlled trial assessing the non-inferiority of counselling for depression versus cognitive-behaviour therapy for patients in primary care meeting a diagnosis of moderate or severe depression (PRaCTICED): Study protocol for a randomised controlled trial

**DOI:** 10.1186/s13063-017-1834-6

**Published:** 2017-03-01

**Authors:** David Saxon, Kate Ashley, Lindsey Bishop-Edwards, Janice Connell, Phillippa Harrison, Sally Ohlsen, Gillian E. Hardy, Stephen Kellett, Clara Mukuria, Toni Mank, Peter Bower, Mike Bradburn, John Brazier, Robert Elliott, Lynne Gabriel, Michael King, Stephen Pilling, Sue Shaw, Glenn Waller, Michael Barkham

**Affiliations:** 1 Health Services Research, Centre for Psychological Services Research, School of Health and Related Research, University of Sheffield, 30 Regent St, Sheffield, S1 2DA UK; 2 Clinical Psychology Unit, Centre for Psychological Services Research, University of Sheffield, Sheffield, S10 2TN UK; 3 Department of Psychology, University of Sheffield, Sheffield, S10 2TN UK; 4 Health Services Research, School of Health and Related Research, University of Sheffield, 30 Regent St, Sheffield, S1 4DA UK; 5 Sheffield IAPT (Sheffield Health & Social Care NHS Foundation Trust), St George’s Community Health Centre, Winter Street, Sheffield, S3 7ND UK; 6 Health Economics and Decision Science, School of Health and Related Research, University of Sheffield, 30 Regent St, Sheffield, S1 2DA UK; 7 NIHR School for Primary Care Research, University of Manchester, Manchester, M13 9PL UK; 8 Clinical Trial Research Unit, School of Health and Related Research, University of Sheffield, 30 Regent St, Sheffield, S1 2DA UK; 9 Counselling Unit, School of Psychological Sciences and Health, University of Strathclyde, Room 507, Graham Hills Building, 40 George Street, Glasgow, G1 1QE UK; 10 School of Psychological and Social Sciences, York St John University, Lord Mayor’s Walk, York, YO31 7EX UK; 11 Division of Psychiatry, Faculty of Brain Sciences, University College London, Sixth Floor, Maple House, 149 Tottenham Court Rd, London, W1T 7NF UK; 12 Research Department of Clinical Health and Educational Psychology, University College London, 1-19 Torrington Place, London, WC1E 7HB UK; 13 c/o Mental Health Group, Health Services Research, School of Health and Related Research, University of Sheffield, 30 Regent St, Sheffield, S1 2DA UK; 14 Department of Psychology, Clinical Psychology Unit, University of Sheffield, Sheffield, S10 2TN UK

**Keywords:** Depression, Non-inferiority trial, Cognitive behaviour therapy, Counselling for depression

## Abstract

**Background:**

NICE guidelines state cognitive behavioural therapy (CBT) is a front-line psychological treatment for people presenting with depression in primary care. Counselling for Depression (CfD), a form of Person-Centred Experiential therapy, is also offered within Improving Access to Psychological Therapies (IAPT) services for moderate depression but its effectiveness for severe depression has not been investigated. A full-scale randomised controlled trial to determine the efficacy and cost-effectiveness of CfD is required.

**Methods:**

PRaCTICED is a two-arm, parallel group, non-inferiority randomised controlled trial comparing CfD against CBT. It is embedded within the local IAPT service using a stepped care service delivery model where CBT and CfD are routinely offered at step 3. Trial inclusion criteria comprise patients aged 18 years or over, wishing to work on their depression, judged to require a step 3 intervention, and meeting an ICD-10 diagnosis of moderate or severe depression. Patients are randomised using a centralised, web-based system to CfD or CBT with each treatment being delivered up to a maximum 20 sessions. Both interventions are manualised with treatment fidelity tested via supervision and random sampling of sessions using adherence/competency scales. The primary outcome measure is the Patient Health Questionnaire-9 collected at baseline, 6 and 12 months. Secondary outcome measures tap depression, generic psychological distress, anxiety, functioning and quality of life. Cost-effectiveness is determined by a patient service receipt questionnaire. Exit interviews are conducted with patients by research assessors blind to treatment allocation. The trial requires 500 patients (250 per arm) to test the non-inferiority hypothesis of −2 PHQ-9 points at the one-sided, 2.5% significance level with 90% power, assuming no underlying difference and a standard deviation of 6.9. The primary analysis will be undertaken on all patients randomised (intent to treat) alongside per-protocol and complier-average causal effect analyses as recommended by the extension to the CONSORT statement for non-inferiority trials.

**Discussion:**

This large-scale trial utilises routinely collected outcome data as well as specific trial data to provide evidence of the comparative efficacy and cost-effectiveness of Counselling for Depression compared with Cognitive Behaviour Therapy as delivered within the UK government’s Improving Access to Psychological Therapies initiative.

**Trial registration:**

Controlled Trials ISRCTN Registry, ISRCTN06461651. Registered on 14 September 2014.

**Electronic supplementary material:**

The online version of this article (doi:10.1186/s13063-017-1834-6) contains supplementary material, which is available to authorized users.

## Background

Since 2008, patients presenting to the UK National Health Service (NHS) with a primary condition of mild, moderate and severe depression are typically treated within Improving Access to Psychological Therapies (IAPT) services. These services are premised on a stepped care model and built on the argument for improved access to, in particular, cognitive behaviour therapy (CBT), which resulted in a large investment in training a new workforce in this particular psychological approach [1]. The model of stepped care within the IAPT initiative required a new workforce of Psychological Wellbeing Practitioners (PWPs) as the immediate point of contact for patients with mild to moderate depression (step 2) with the aim of their providing psycho-educational interventions with the option of stepping up patients to high-intensity CBT therapists (step 3) should initial benefits to patients not be realised. An initial implementation of the stepped care model was carried out at two demonstration sites [2, 3] followed by an expansion to 32 pathfinder sites [4] and then by national rollout.

To date, the IAPT programme has yielded updates from the Department of Heath [5] as well as evaluations of differing aspects of the implementation [6]. While the implementation of IAPT at step 2 and step 3 originally focused only on training and delivering CBT-based personnel and interventions, more latterly the provision of psychological approaches at step 3 has been extended to include bona fide psychological therapies in addition to CBT, namely interpersonal psychotherapy, dynamic interpersonal therapy, couples counselling, and counselling for depression (CfD), with the latter being the focus of the present article.

The NICE review of psychological interventions for depression identified CBT as the front-line psychological intervention while counselling was assigned to situations in which first-line interventions were either not successful or were not preferred by the patient on the basis that the evidence for counselling was ‘uncertain’ [7]. The robust evidence base for CBT was a key factor in the UK government’s funding of the IAPT initiative and the drive to train large numbers of practitioners in CBT as part of workforce development in Primary Care. While CfD is, therefore, one of the NICE-recommended psychological therapies for mild to moderate depression made available within IAPT services, its role is secondary to CBT and there is no evidence underpinning its implementation for patients presenting with more severe levels of depression.

This article sets out the protocol for a randomised controlled trial embedded within one IAPT service to determine the relative efficacy of CfD as compared with CBT. The protocol adheres to the Standard Protocol Items: Recommendations for Intervention Trials (SPIRIT) checklist, which is available as an Additional file 1.

### Review of existing literature

A review of six RCTs showed patients who were assigned to counselling demonstrated a significantly greater reduction in psychological symptoms such as anxiety and depression than patients receiving usual GP care when followed up at up to 6 months [8]. These psychological benefits were modest: the average counselled patient was better off than approximately 60% of patients in usual GP care. However, there were no significant differences between counselling and usual care in the four RCTs reporting longer-term outcomes (8 to 12 months).

A trial comparing non-directive counselling with CBT yielded similar outcomes amongst the two therapies in their overall effectiveness at short- or long-term follow-up [9]. Both therapies were superior to usual GP care in the short term but provided no significant advantage in the long term. Findings from this trial were not included in the Depression Guidelines because of a significant proportion of patients having a diagnosis of mixed anxiety and depression. However, a subsequent re-analysis of data focusing only on those patients meeting a diagnosis of depression confirmed the earlier results [10].

In a meta-analysis, a comparison of CBT with therapy similar to counselling (non-directive supportive therapy) demonstrated no statistically or clinically significant difference with a small advantage in favour of CBT of d = 0.05 (95% CI −0.08, 0.18) [11]. However, the authors commented that this difference is small, its clinical relevance is unclear, and the collection of studies included under the broad heading of supportive psychotherapy may have been overly heterogeneous. Further, CBT had the highest relative risk of drop out (k = 26, RR = 1.16).

A state-of-the art review of the literature regarding person-centred and experiential therapies reported that Person-Centred Therapy (PCT) appeared to be consistently, statistically and practically equivalent in effectiveness to CBT (22 studies, including 17 RCTs, with effect sizes of −0.06 and −0.1 respectively [12]. Further, evidence from practice-based studies indicates that PCT, as defined by the practitioners and as delivered in the NHS, is effective and not significantly different from CBT [13, 14]. In response to the IAPT initiative, a recent review of data from the 1-year rollout indicated that for depression, counselling was as effective as CBT [6].

In terms of psychological approaches taken up by adults experiencing specified levels of depression within the past week, data released by NHS Digital from the 2014 Office of National Statistics (ONS) Adult Psychiatric Morbidity Survey (APMS) identified counselling (including bereavement counselling) as the most used psychological intervention (7.7%) followed by psychotherapy (7.2%) and CBT (5.6%) [15]. For those adults meeting a specified criterion of severity (score 18+) on the Clinical Interview Schedule-Revised [16], the rates for being in receipt of psychological therapies was highest for CBT (6.5%), followed by counselling (5.7%) and then psychotherapy (4.5%). These data indicate that generic counselling is a prominent psychological intervention and that it is also being delivered to adults presenting with more severe levels of depression. Recently published data for 12 months (2015–16) from the UK national IAPT programme reports utilisation rates of 152,452 for CBT and 61,414 for CfD. The recovery rates for patients completing a course of treatment for depression at step 3 (high intensity) were 45.9 per cent for CBT and 47.6 per cent for Counselling for Depression (CfD) [17].

Hence, the collective evidence from a number of sources suggests either small differences or broadly equivalent results when making comparisons between CBT and counselling (as a broad discipline) as well as with CfD specifically. However, these results may be due to a number of factors, for example the heterogeneity of non-CBT comparators and the over-sampling of mild depression. There is a recent small pilot feasibility comparison between nondirective counselling (not CfD) and CBT for persistent sub-threshold mild depression that obtained no difference in findings; however, this is probably too narrow a severity band to be relevant to typical depressed populations encountered in counselling and its associated practitioners [18].

However, there is no robust trial evidence supporting the use of counselling and, more specifically, CfD with severe depression. Accordingly, there is a need for a randomised controlled trial of CfD for moderate and severe depression. Furthermore, given that CBT is the current treatment of choice for moderate and severe depression, there is a need to know the relative efficacy of CfD as compared with CBT (rather than, for example, a no-treatment condition). Hence, it is important that all stakeholders have access to better quality evidence concerning the efficacy and efficiency of Counselling for Depression (CfD). Furthermore, in terms of ensuring that patients have a choice of differing talking therapies, establishing the efficacy of CfD is important.

### Pilot work and determining type of trial

In light of the extant literature, there was no basis for adopting a superiority trial. Analyses of existing Sheffield IAPT service data (1 April 2009–30 September 2010) indicated only small differences in outcomes between CBT and counselling. In the analysis of patients with PHQ-9 intake scores ≥12 in the Sheffield service data, the overall mean (SD) pre-last change in PHQ-9 was 6.8 (6.9) and there was no significant difference between counselling and CBT (difference = +0.5 points on the PHQ-9 in favour of counselling; 95% CI −0.3, +1.3). Analysis of a further data set from the same service of data collected between June 2010 and October 2013 showed a small effect size advantage to CBT of 0.16 with the extent of pre-post change being 1.0 PHQ-9 point greater for CBT (7.3) than counselling (6.3) [19]. When number of sessions and type of therapy ending were entered into the multilevel modelling, treatment modality was not significant.

From these findings, we predicted the actual difference in change means between approaches to be close to zero. However, we were mindful that these data were derived from counselling as delivered in a routine practice setting and not from CfD. In addition, the aim of the trial was to underpin the delivery of CfD within the IAPT service delivery system as a viable alternative to CBT. Accordingly, we proposed a pragmatic trial and reasoned that the primary aim of the trial was to test that CfD as delivered in routine settings was non-inferior to CBT within an agreed *a priori* tolerance. The central tenet of a non-inferiority trial is that the candidate treatment does not yield patient outcomes that are inferior to a benchmark treatment such that would be clinically notable. Accordingly, we proposed a non-inferiority trial.

### Objectives

The primary objective is to determine the clinical and cost-effectiveness of CfD compared with CBT as delivered in primary care for patients presenting with moderate or severe depression. The secondary aims are to explore patients’ experiences of the treatments received and, for those patients who drop out of treatment, to gather information as to the reasons. The outcomes of trial patients will also be compared to patients within the IAPT service but who were not participants in the trial.

## Methods

### Design

The PRaCTICED trial is a pragmatic, two-arm, parallel group, non-inferiority RCT comparing the clinical efficacy and cost-effectiveness of CfD and CBT within a local Improving Access to Psychological Therapies (IAPT) service. The trial utilises all mandated data collected routinely as part of the IAPT service as well as additional data required by the trial design (see later for details). This reported version of the protocol conforms to the Standard Protocol Items: Recommendations for Interventional Trials (SPIRIT) guidelines. A copy of the SPIRIT Checklist is contained as part of the supplemental materials (see Additional file 1).

### Participants/inclusion and exclusion criteria

Participants are patients receiving step 2 treatment within the IAPT service in Sheffield, UK, and who meet the following inclusion criteria: aged 18 or over and having been deemed to require stepping up by a Psychological Wellbeing Practitioner (PWP), a score of 12 or more on the PHQ-9, with depression as their major focus for treatment. Patients meeting these general criteria are invited to a screening assessment to determine their eligibility for the trial. An initial criterion is that patients do not have a strong preference such that they would be unwilling to accept one of the treatments if they were randomised to it. This is checked by PWPs and verified at the screening interview.

Other exclusion criteria are: presence of organic condition, psychosis, drug or alcohol dependence, or elevated clinical risk. Patients may be in receipt of medication for depression but the regime must be stable at the point of entry to the trial. If they are in receipt of medication, this will be recorded.

### Study setting

The study is embedded within the Sheffield IAPT services, covering potentially 93 GP practices. The population of Sheffield is 560,000 people and the city region has an Index of Multiple Deprivation of 17.9% placing it as seventh most deprived core city in England. It is ranked 60th out of 326 in terms of most deprived local authorities in England and nearly one quarter of the Lower Super Output Areas are within the most deprived 10% nationally.

The Sheffield IAPT service comprises four distinct geographical sectors: southeast, southwest, north and west. It routinely delivers both counselling and CfD within the step 3 service as well as CBT to patients presenting with depression who have not responded to a low-intensity treatment (step 2 in the IAPT stepped care model). CfD counsellors and CBT therapists undertake their IAPT work within GP practices or at a central location thereby ensuring that accessibility for patients receiving treatment in each locality of Sheffield is optimal.

### Psychological interventions

The psychological intervention being evaluated is CfD as the candidate intervention against Beckian CBT, which is acting as the comparator benchmark intervention. Both treatments are currently offered as standard within the IAPT service.

#### Counselling for Depression (CfD)

CfD [20, 21] is a form of person-centred/experiential (PCE) therapy derived from the competences required to deliver effective humanistic psychological therapies for depression. CfD is drawn from those humanistic approaches with the strongest evidence for efficacy, based on outcomes of controlled trials (for a review, see [12]). CfD is specifically designed to address depression and is delivered within IAPT and related programmes. Whilst counselling has long been available in NHS Primary Care settings, service design and treatment approaches in practice have proved very variable.

The CfD curriculum was developed by BACP [the British Association for Counselling and Psychotherapy, sponsored by the UK Department of Health (DH)] and the work of the design team informs this protocol. The programme trains counsellors to provide a depression-specific therapy for individual patients (in an IAPT setting where a patient has not responded to low-intensity intervention or actively opts for counselling). The CfD competences are outlined in an IAPT-endorsed framework drawn from a number of NICE-endorsed research studies and from key texts identified by the Humanistic Psychological Therapies Expert Reference Group that describe the modality and underpin its effectiveness [22]. Person-centred counselling [23] and emotion-focused therapy [24] have much in common both theoretically and in terms of their methods. When used in combination they are often referred to as person-centred/experiential therapy.

Prior to the trial commencing, we provided CfD training to all counsellors in Sheffield IAPT that has facilitated a move towards standardised practice and evidence-based service evaluation. CfD training standardises counselling work with depressed patients and aligns therapist interventions with the evidence-base underpinning NICE guidelines. The CfD training is aimed at experienced person-centred and humanistic practitioners as a ‘top-up’ provision. The training consisted of a 5-day taught programme delivered across a 1 or 2-week block, followed by a period of supervised clinical work. During clinical practice associated with CfD training, a minimum of 80 h of supervised practice must be completed. Only those who have completed the training will be included within the trial, meaning increasing numbers of counsellors will be included as the trial progresses. The delivery of CfD is standardised by adoption of the text *Counselling for depression: A person-centred and experiential approach to practice* [25]. Manuals based on this text, the CfD theoretical approach and training have been developed and provided to all counsellors to act as an on-going reference and training resource [26].

#### Beckian Cognitive Behavioural Therapy (CBT)

The comparator is high-intensity CBT as delivered within the Sheffield IAPT service. The curriculum for high intensity CBT states that CBT is now known to be an effective treatment option for many problems. In the NICE guidelines for anxiety disorders and depression CBT was strongly recommended [7].

CBT within the IAPT service comprises two protocol driven interventions: Beckian cognitive therapy [27, 28] and Martell’s behavioural activation [29]. These interventions are delivered by high-intensity CBT practitioners in accordance with NICE guidance in which CBT and BA are recommended for the treatment of mild to moderate depression but only CBT for the treatment of severe depression. Although the COBRA trial addressed the comparative efficacy of BA versus CBT for depression [30] the comparator treatment in this trial will be confined to CBT only so as to ensure clarity of the comparator and to maximise comparison with other trial evidence using CBT. Representing equal commitment to the comparator treatment, we provide regular ‘top-up’ workshops for all Sheffield IAPT CBT practitioners, so that all practitioners receive up-to-date training in their respective treatment method prior to and during the trial.

The delivery of CBT is standardised by the adoption of the text *Cognitive behaviour therapy: Basics and beyond* (2nd edition) [28], which is available to all CBT practitioners supporting the trial. In addition, a CBT Manual has been written, termed a Clinical Practice Guide (CPG), to guide the delivery of CBT in the trial [31]. This has been based on a similar CPM written for two recent major UK trials of CBT: CoBaLT and COBRA [32]. The CPM has been adapted and developed with input from trial co-applicants (SK & GW) and the lead CBT practitioner in the Sheffield IAPT service. It does not present any new component of CBT but simply acts as a reminder to all practitioners to adhere to the treatment model being delivered.

### Treatment delivery

Patients are offered a maximum of 20 sessions in either intervention as this is the maximum number stated for CBT and for CfD. Accordingly the potential maximum course of the interventions is similar for both interventions. Patients only discontinue in their assigned intervention if the therapist, following discussion with their supervisor, considers there are strong clinical grounds for doing so.

### Training in the psychological models

The counsellors are required to complete 80 h CfD experience in four 20-h blocks with these sessions being audio-taped. It is standard practice within the IAPT model of competencies and within Sheffield IAPT for counsellors (and CBT therapists) to audio-record sessions to receive quality supervision. This is consistent with the CfD national curriculum.

Practitioners select one tape from each of the four blocks of 20 tapes submitted to the expert trainers to be assessed on a developmental trajectory. The final tape assessment determines their competency as a CfD practitioner. These standards are set out in the IAPT national document and are, therefore, national standards that are expected for any person working as a CfD practitioner.

The CBT practitioners all meet the IAPT training standards. However, they will be provided with additional training directed to ensuring that their delivery is consistent with Beckian CBT. Half-day workshops will be delivered for trial therapists focusing on CBT treatment of depression and will be led by local experts in CBT.

### Clinical supervision and adherence/competency monitoring

We will monitor and assess adherence and competence through two methods: clinical supervision and rating of audiotapes of therapy sessions. The benchmark adherence/competence rating scales for each therapy condition will be used: for CfD, the Person Centred and Experiential Psychotherapy Rating Scale (PCEPS) [33] and, for CBT, the Cognitive Therapy Scale-Revised (CTS-R) [34, 35].

Clinical supervision is carried out as standard in line with IAPT guidelines, ensuring that only qualified CfD supervisors supervise CfD-trained counsellors. Supervisors use a simplified four-item version of the Person Centred and Experiential Psychotherapy Scale (PCEPS) to monitor adherence together with general competency at their supervision sessions during the course of the trial. For any one patient, this is carried out at sessions 2, 6 and 12 (should the patient receive that number of sessions). This procedure ensures that adherence and competency data are available for all patients in the trial. CBT supervision mirrors the CfD process using a simplified four-item version of the Cognitive Therapy Scale-Revised (CTS-R) completed by the supervisor at sessions 2, 6 and 12. These session forms are referred to as the Session Adherence and Competence Scale (SACS) for CfD (SACS-CfD) and for CBT (SACS-CBT).

### Treatment fidelity

Regarding assessing treatment fidelity, our strategy is to ensure that tapes from each practitioner are sampled to establish that each treatment arm is being delivered according to the specified standard. The procedures for assessing treatment fidelity will be identical for both interventions. The description here applies to each intervention arm. The raters for the two interventions will be independent to minimise contamination.

- *Stage 1 (Calibration)*: A sample of five tapes (one from five practitioners selected at random) for each intervention will be rated by national experts to provide a target rating to be used in the training and standardisation of subsequent ratings. The national rater(s) for CfD will be based at the University of York St John and for CBT at the Oxford Cognitive Therapy Centre (OCTC).
- *Stage 2 (Independent fidelity ratings)*: Digital recordings of sessions will be selected at random using the following procedure. At the therapist level, for each therapist, one case will be selected at random per block of five seen cases (or upwards of 5). Hence, the sampling strategy ensures that (1) all therapists are sampled and (2) the pool of rated tapes and overall competence ratings reflect the differential loading carried by therapists. At the session level, for each case sampled, the selected session will be randomly selected from early (excluding session 1), middle or late (excluding final session). This sampling strategy will yield a total of 50 tapes per intervention, thereby providing a total of approximately 100 tapes to be rated in the trial (although the actual number may vary as a function of numbers of therapists and the number of sessions delivered).
- *Stage 3 (Independent fidelity audit)*: As a final check, a small subsample of the independent fidelity ratings will be audited by experts in the respective therapies.

### Patient consent process

PWPs are the initial point of contact and gatekeepers for entry into the trial (i.e., there is no direct GP referral or self-referral into the trial). PWPs utilise both face-to-face as well as telephone assessments of patients in their work. If the PWP considers that, during their initial assessment of the patient or their subsequent work with them, the trial would be an appropriate course of treatment, then they introduce the trial and request consent for the research team to contact them. Appropriateness for the trial is that a patient’s PHQ-9 score is 12 or higher and that they present with depression and wish to be treated for depression. Ethics approval has also been given for this procedure to be carried out over the telephone. Potential participants are then sent information on the trial and a Consent to Treatment form together with an appointment date for an assessment interview.

The PWP contacts the patient 2–4 days before the screening appointment to answer any questions about the trial and check that the patient is still willing to attend the screening. Should the patient not wish to proceed with the trial, the PWP will carry out the usual procedures for non-trial patients. At the screening appointment, the patient has the opportunity to ask any further questions before signing consent forms to enter the trial. They are also provided with contact information and consent to be contacted by researchers in the future.

### Diagnostic assessment

**Fig. 1:**
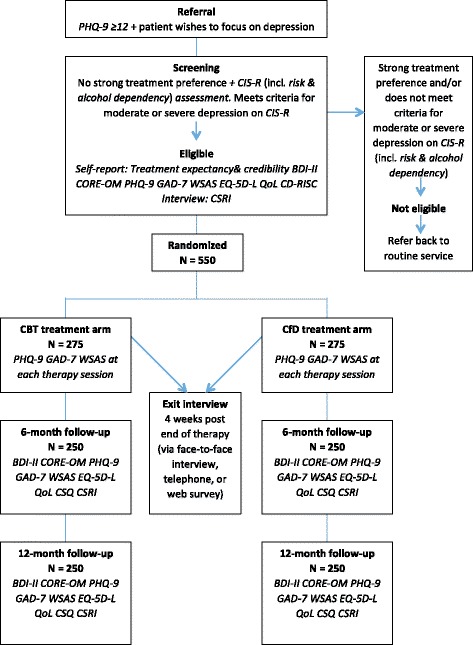
Study flow diagram of referral, screening and allocation of patients to the PRaCTICED trial. Legend: *BDI-II* Beck Depression Inventory II, *CSQ* Client Satisfaction Questionnaire, *CD-RISC* Connor-Davidson Resilience Scale, *CIS-R* Clinical Interview Schedule-Revised, *CSRI* Client Service Receipt Inventory, *CORE-OM* Clinical Outcomes in Routine Evaluation-Outcome Measure, *EQ-5D-5L* Euroqol 5D-5L, *GAD-7* Generalised Anxiety Disorder-7, *MINI* Mini International Neuropsychiatric Interview, *PHQ-9* Patient Health Questionnaire-9, *QoLS* Quality of Life Scale, *WSAS*, Work and Social Adjustment Scale

Patients attending the assessment interview are asked again whether they have a strong preference for one or the other treatment such that they would refuse a treatment if it were offered to them. The primary measure for determining suitability for the trial is the Clinical Interview Schedule-Revised (CIS-R) with the requirement that patients meet an ICD-10 diagnosis of moderate or severe depression [16]. Assessment interviews are carried out either by Clinical Support Officers (CSOs) or members of the research team. All assessors are trained in using the CIS-R. If active thoughts of suicide are indicated from the CIS-R, we implement a risk protocol to inform the PWP or identified practitioner. In terms of alcohol or substance dependency, these are determined by specific questions from Section I (Alcohol) and Section II (Drug) of the Mini-International Neuropsychiatric Interview (M.I.N.I.) [36], which yield diagnoses of current alcohol or drug dependency. If a patient meets an ICD-10 diagnosis of moderate or severe depression, they are consented into the trial. Patients who do not meet the criterion are talked through the reasons and referred back to the PWP. Figure 1 presents a flow study chart of the progress of patients through the trial.

### Randomisation

Consenting patients are allocated to one of the two treatment arms via remote access to the randomisation procedure that is hosted by epiGenesys, a wholly owned subsidiary of the University of Sheffield.

### Blinding

Allocation to either intervention is recorded in a separate location in the patient data log. The assessors carrying out the therapy exit interviews do not have immediate access to information regarding allocation assignment. Statisticians conducting the analysis will not be involved in the administration of the trial and will be blinded to the randomisation. Key variables (i.e., treatment assignment) will be coded as non-identifiable variables to minimise potential biasing in analyses. Blinding of a patient’s assignment to treatment will only be unmasked on the specific direction of the Data Management and Ethics Committee.

### Measures

**Fig. 2:**
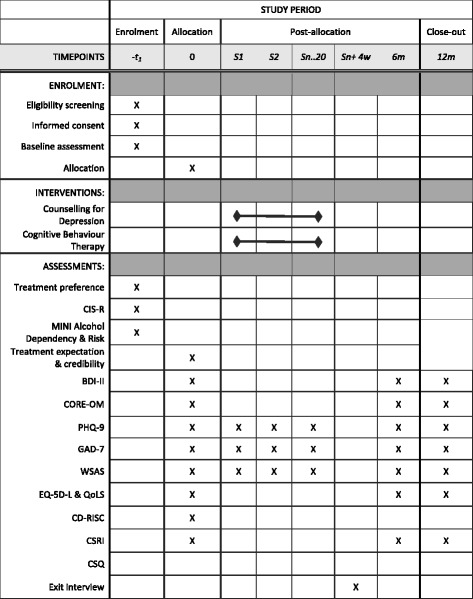
SPIRIT diagram of assessments at enrolment, allocation, weekly sessions, and 6- and 12-month time points Legend: *BDI-II* Beck Depression Inventory II, *CD-RISC* Connor-Davidson Resilience Scale, *CIS-R* Clinical Interview Schedule-Revised, *CSRI* Client Service Receipt Inventory, *CSQ* Client Satisfaction Questionnaire, *CORE-OM* Clinical Outcomes in Routine Evaluation-Outcome Measure, *EQ-5D-5L* Euroqol 5D-5L, *GAD-7* Generalised Anxiety Disorder-7, *MINI* Mini International Neuropsychiatric Interview, *PHQ-9* Patient Health Questionnaire-9, *QoLS* Quality of Life Scale, *WSAS*, Work and Social Adjustment Scale

Patients then complete the second part of the assessment interview comprising the completion of a battery of measures. All assessors receive a standard training regarding the collection of assessment data. A summary of the assessments is presented in Fig. 2.

#### Treatment Preferences and Credibility

Patient preferences and credibility regarding the two interventions are measured using standard scales adapted to the specific interventions [37, 38].

#### Primary Outcome

##### Patient Health Questionnaire-9 (PHQ-9) [39]

The primary outcome measure is the PHQ-9, a brief (9-item) self-report 4-point Likert-type scale measure of depression. Items correspond to each of the nine DSM-5 criteria for depression [40]. Items ask patients to rate how often they have been affected by symptoms (of depression) over a 2-week time period prior to completing the questionnaire.

Individual item scores range from 0 (*“not at all”*) to 3 (*“Nearly every day”*) with total PHQ-9 scores ranging from 0 to 27. Scores of 10 and above are demarcated as clinical scores. Scores of 5–9, 10–14, 15–19 and 20–27 are classified as reflecting mild, moderate, moderately severe and severe levels of depression respectively. The measure has an internal reliability of 0.89 and a test-retest reliability of 0.84 across 48 h. The PHQ-9 is mandated by the IAPT service and is completed by patients at each session attended along with other mandated measures within the IAPT minimum data set (MDS) [41].

#### Secondary Outcome Measures

A range of secondary outcome measures is collected. At baseline, the intensity of depression is measured via the Beck Depression Inventory-II (BDI–II) [42], psychological distress via the Clinical Outcomes in Routine Evaluation-Outcome Measure (CORE-OM) [43], generalised anxiety via the Generalised Anxiety Disorder Assessment (GAD-7) [44], plus the Work and Social Adjustment Scale (WSAS) [45]. Mobility, self-care, usual activities, pain/discomfort and anxiety/depression are measured via the EQ-5D-5L [46, 47] and Quality of Life Visual Analogue Scale (QoLS) (personal communication: J. Connell). In addition, patients complete the Connor-Davidson Resilience Scale (CD-RISC) [48]. Patient service receipt data are also collected [49]. At 6- and 12-month follow-ups, in addition to the PHQ-9 as the primary outcome, the BDI-II, CORE-OM, GAD-7, WSAS, EQ-5D-5L, QoL and CSRI are collected, as is the Client Satisfaction Questionnaire (CSQ) [50].

Four weeks after a patient ends treatment, either through completing or dropping out from sessions, they are contacted to undertake a brief questionnaire/short interview (telephone, face-to-face or web-based) to collect data on their experiences of the treatments.

### Sample Size

Published findings [2–4] and Sheffield IAPT service data (1/4/09 – 30/9/10) indicate only small differences in outcomes between CBT and counselling. Hence, from these findings we predict the actual difference in mean change between treatments to be zero. Accordingly, the margin within which CBT could not be considered statistically or clinically more effective than CfD was determined as follows: First, treatment effects of 0.2 to 0.3 are conventionally viewed as ‘small’ and of limited clinical value (e.g., [51, 52]). Second, it has been recommended that the threshold for non-inferiority be set at 50% or less than the expected difference between CBT and usual care, which would mean an effect size of less than 0.3 (i.e., 0.6/2). Finally, discussions with psychologists on the research team and IAPT staff indicated that less than 2 points on the PHQ-9 is not perceived as clinically important, which is equivalent to an effect size of just under 0.3 (given the pre-last SD of 6.9 found in the service data above). Therefore, a pre-last change difference of less than 2 on PHQ-9 in favour of CBT was adopted as the limit for non-inferiority of CfD.

It is estimated that 550 patients (275 per arm) would need to be recruited to the trial to secure the 500 patients needed to test the non-inferiority hypothesis at the one-sided 2.5% significance level with a power of 90%. This assumes a standard deviation of 6.9 (derived from the aforementioned service use data, which incorporate both inter-patient and inter-therapist variability), no underlying difference between the effect of CBT and counselling and a 10% loss to 6-month follow-up. As the trial is within a service with few additions to routine practices and procedures, it is expected that relatively few participants will leave the trial and not provide a 6-month follow-up PHQ-9. To sustain adequate referrals via PWPs, weekly monitoring of referral rates in each sector occurs together with monthly feedback via sector managers as well as target setting.

### Data management

Data entry will be checked using a 5% double-entry procedure. Anonymised measures data are stored electronically on a University of Sheffield password-protected secure server, with only named people having access. No patient personal details are stored electronically. Paper copies of measures are stored in locked filling cabinets behind two locked doors. Patient contact information, on paper, is stored in different locked filling cabinets, in a different office, again behind two locked doors. The trial adheres to the University of Sheffield Ethics Policy Governing Research Involving Human Participants, Personal Data and Human Tissue (version 6). Information Governance toolkit details for electronic data storage are as follows: organisation code 8D715-SHR, version 13, 2015/16; reviewed grade satisfactory (level 2 or above).

### Statistical methods

Statistical analyses will be carried out by a statistician and senior statistician from the Clinical Trials Research Unit (CTRU). Neither will be involved in the administration of the trial and both will be blinded to the randomisation and assignment. The two treatment arms will be coded as non-identifiable variables to minimise potential biasing in analyses.

### Primary analysis

The primary analysis will adhere to a Statistical Analysis Plan (SAP) devised by an independent statistician under the guidance of the senior medical statistician. The SAP was informed by the International Conference on Harmonisation topic E9 [53], reference to the literature (e.g., [54]) and applicable standard operating procedures from the University of Sheffield Clinical Trials Research Unit (CTRU). Non-inferiority of counselling to CBT will be concluded if the CI lies entirely above the non-inferiority limit of −2 units (i.e., that a difference as large as 2 units in favour of CBT has been ruled out).

In contrast to superiority trials, the ITT analysis is anti-conservative (i.e., it underestimates the treatment effect) when looking at non-inferiority. Specifically, if an intervention is not delivered as fully planned by the protocol the ITT analysis dilutes the treatment difference and therefore raises the risk of having the groups look artificially similar (and hence CfD being artificially non-inferior to CBT). Given the pragmatic nature of the trial the ITT will remain the primary analysis population, but additional consideration will be given to other analyses that exclude participants who did not receive the intervention as planned. It should also be noted there is a general lack of guidance regarding the choice of the primary analysis population for non-inferiority trials [55].

The additional analysis populations are:

1. An objective per-protocol analysis (PP1) that excludes participants who receive fewer than four sessions within 6 months of randomisation and/or who switch treatment arms within 6 months of randomisation.
2. A case-review per-protocol analysis (PP2) in which participants are assessed on a case-by-case basis for the number and timing of sessions, additional therapies received and therapist adherence to principles of CBT or CfD. This will necessarily be unblind to treatment group but will be blind to outcome data.
3. Complier-adjusted causal effect (CACE) models in which participants who undergo their intervention in accordance with protocol are compared to those in the comparator group who are “likely” to have done so (based on statistical modelling) had they been randomised to receive it. Two CACE analyses will be undertaken, one of which excludes “non-receivers” of CBT and the second removing “non-receivers” of CfD.

A fuller description of these analyses is provided in the Statistical Analysis Plan.

### Secondary analysis

This will consider baseline to 6-month and baseline to 12-month change in PHQ-9, BDI-II, CORE-OM, GAD-7, WSAS, and EQ-5D-5L and QoLS using the same methodology as for the primary outcome measure. Similarly, change from baseline to the routinely collected end of therapy score on PHQ-9, GAD-7 and WSAS will be analysed. The proportions of patients making reliable and clinically significant change [56, 57] on PHQ-9, BDI-II, CORE-OM and GAD-7 will also be compared. Additional exploratory analyses will be used to identify characteristics of patients and therapists that are predictive of better outcomes overall and within each therapy. In addition, the reasons why patients leave therapy prematurely and the experiences of patients who remain in or leave therapy will be investigated. Consideration will also be given to the number and effect on outcome of patients experiencing sudden gains in each treatment arm.

### Routine service data for patient cohort

For the period spanning patient recruitment into the trial (approximately 36 months), all patients who are stepped up to step 3 will define the patient cohort within which the trial is embedded. Data collected routinely as part of the service for non-trial participants will be made available in anonymised form as a comparator. These data will provide added value in terms of external validity and will allow comparisons to be made between trial participants and non-participants, in order to consider the representativeness of our research sample. The ability to derive this comparison addresses a key limitation of trials methodology in terms of external validity.

Given that the primary outcome measure is standard throughout IAPT services, the outcomes of trial and non-trial patients can be compared with those from published literature on counselling and CBT within routine IAPT services [2–4, 58]. This approach does not place any additional burden on non-trial participants, as the measures they complete are routine and mandatory as part of the IAPT service agreement. Further, it does not add cost to the proposed study and therefore is clear added value. In addition, as the data will contain sessional PHQ-9 scores including a last session attended (end of therapy) score, it will be used in conjunction with trial data for further analyses.

### Missing data

By recruiting sufficient numbers to account for trial dropout to 6-month follow-up, it is planned that primary endpoint data will be adequate to address the main research question. Routinely collected PHQ-9 scores will be available for sessions attended prior to dropout, and these will be used as part of the imputation process where the 6-month endpoint data are missing. It is expected that 80% of patients who have competed treatment will provide research data at 12 months post-randomisation. Isolated instances of missing data will be imputed by linear interpolation. Multiple imputation methods will be used for patients with more substantial missing data, and the sensitivity of the results will be further assessed by imputing alternative values based on the reason for dropout.

### Economic analysis

We will establish the cost-effectiveness of CfD compared to CBT [59]. The purpose is to establish what the additional benefit and resource implications of CfD are relative to CBT. The primary analysis will be from a health and social care perspective and will therefore include costs to the NHS and social care services. The method used to conduct this economic analysis will depend on treatment outcomes.

Where treatment outcomes are found to be equivalent based on the primary measure of efficacy, a cost minimisation analysis will be conducted. In this case, the focus will be in assessing any cost differences between CfD and CBT.

Total costs of each intervention will be estimated using the number of sessions multiplied by national unit costs and data from the local Trust. The consequences for the use of other health and social care resources (including hospital admissions, outpatient attendance, GP visits, other therapy and medication) will be measured using a patient-completed resource use questionnaire and service data and costed with national unit costs. Individual level mean costs (intervention and other resource use) for CfD and CBT will be compared; uncertainty around the costs estimates will be generated using probability sensitivity analysis. One-way sensitivity analysis will be conducted on key assumptions such as the number of sessions.

Where one intervention proves to be more effective, then a cost-effectiveness analysis (CEA) will be undertaken using the estimated incremental cost per quality adjusted life year (QALY), that is, the difference in outcomes divided by the difference in costs for CfD and CBT. The primary outcome measure for the CEA will be the EQ-5D-5L. The EQ-5D-5L is a generic preference-based measure of health designed for calculating QALYs. It is composed of five dimensions: mobility, self-care, usual activities, pain/discomfort and anxiety/depression, each with five levels describing 3125 health states in total. The EQ-5D-5L is a revision of the EQ-5D, the NICE recommended measure for economic evaluation, offering better sensitivity [46]. The EQ-5D-5L has been valued by the general public in England and we will apply these new values to generate utility values [60]. QALYs will be estimated from the EQ-5D-5L collected from patients at baseline, 6 and 12 months. Individual patient level data on costs and QALYs over 12 months will be used to estimate the mean cost-effectiveness of CfD compared to CBT and the underlying uncertainty around it by a probabilistic sensitivity analysis.

One-way analysis of key assumptions will be undertaken and where differences persist at 12 months then the analysis will be extrapolated beyond 12 months. Additional analysis will include assessing outcomes for CfD compared to CBT in terms of the proportion who achieve reliable and clinically significant improvement based on the PHQ-9 [56, 57]. Patients will be classified as having had a reliable and clinically significant improvement if they change by 6 points *and* move from a clinical population at baseline (10 and above) to a non-clinical population (9 or less) at 12 months. This will be combined with incremental costs to establish the incremental costs associated with reliable and clinically significant improvements.

### Process Analyses

To achieve a fuller understanding of patients’ experiences of CBT and CfD and how these impact of the course of treatment, we will carry out a programme of process studies using a defined sampling frame comprising cases of therapy non-completion and accounts of positive change (based on questionnaire returns and selective interviews); patient engagement, resilience and therapeutic alliance (based on a subsample of routinely collected tapes); and the phenomenon of sudden gains and deteriorations (based on routinely collected sessional PHQ-9 scores). We will also be investigating therapy non-completion and accounts of change through telephone or face-to-face interviews with consenting participants who have dropped out of therapy.

### Risk procedures and reporting of adverse events

We follow standard operating procedures in relation to assessing patient risk and reporting and acting upon serious adverse events. All associated research staff are trained in the recognition of and response to distress and risk. Written protocols are followed, based on the standard operating procedures of the Clinical Trials Research Unit (CTRU), which are consistent with the Trust procedures within which framework we work.

### Public and patient involvement panel

A panel of service users and people with lived experience—the Public and Patient Involvement (PPI) Panel—has been established to advise on the strategy, implementation and future analysis of the data. One of the grant holders is a person with lived experience. Members of the PPI panel have been co-opted onto the Trials Steering Committee as well as the Trials Management Group. The panel is informed by recent guidance on PPI involvement [61]. The PPI panel meets approximately every 4 months to discuss issues arising from the implementation of the trial. Views and suggestions are fed back into the TSC and TMG agendas as a standing item. The PPI involvement is being extended to include patients who have completed the trial and who are able to provide input and feedback on its potential benefits.

### Confidentiality

Names of participants on the consent forms are stored separately from data in locked filing cabinets and only accessible by named personnel. A separate key linking names to ID numbers used in data files is stored in a password-protected file on a secure server and only accessible by named personnel.

### Governance and oversight of the trial

A Trials Steering Committee (TSC) has been established with an independent Chair and comprises representatives from differing professional groups together with members of the research team. A Data Monitoring and Ethics Committee (DMEC) has been convened under an independent chair with statistical expertise together with two independent clinicians. A data analyst independent of the trial provides closed reports to the DMEC for scrutiny of adverse events and any trends in the outcomes that would suggest it to be unethical to continue the trial. The chair of the DMEC provides a closed report to the Chair of the TSC. Any decision to stop the trial rests with the independent members of the TSC. In addition, a Trial Management Group (TMG) has been established comprising the research team and key local personnel involved in the trial with the purpose of checking operational procedures. The TMG is chaired by the lead investigator. A site file is maintained and constantly updated with all associated documentation.

## Discussion

The PRaCTICED trial is a large-scale (and may be the only) randomised trial investigating the efficacy of CfD against CBT. Extensive evidence exists for the efficacy of CBT but the evidence for other psychological therapies lags behind. Determining the relative efficacy of other bona fide therapies is important for all stakeholders. The non-inferiority design adopts a realistic approach to such a comparison in the context of a pragmatic trial nested within an existing NHS IAPT service. Advantages of an embedded trial include having access to routinely collected outcome measures as well as the additional measures required specifically by the trial. The routinely collected data include weekly-administered outcome measures, including the primary outcome measure (PHQ-9). Not only does this increase the likely response rate in terms of the primary outcome, but it also provides the additional level of repeated weekly measurement on the primary outcome measure across the course of treatment. Hence, the data collection demand on trial patients is only proportionately greater than for non-trial patients receiving therapies within the routine IAPT service. Further additional advantages of the trial utilising a routine service lies in having access to larger number of therapists. While the exact total number will not be known until the trial is completed, the aspiration is to have in the region of 30 therapists in total delivering interventions in the trial. As such, this will provide a minimum level in order to be able to investigate therapist effects.

However, carrying out trials within routine service settings has considerable challenges. One specific challenge has been the scaling up of sufficient numbers of counsellors trained in the CfD model. Given the part-time nature of the profession, training has been slow. In addition, attempting to train all existing counsellors in the service assumed that they would all embrace the CfD model, an assumption that has had to be adjusted (unpublished observations: A. Nye, J. Connell, M. Barkham). In terms of the availability of both interventions at GP surgeries, this has proved to be a challenge. However, the issue has been largely resolved by the addition of a central wait list resourced by available trial therapists [62]. At an organisational level, the requirement for prioritising and implementing a rigorous trial amidst the realities of an NHS IAPT service delivering therapies under ever-increasing national performance targets and annual cost efficiencies is a major challenge. This has necessitated generating innovative and flexible partnership models between the research team and service organisation. For example, a key decision has been the part-time secondment of a PWP working in the service to the trial research team, thereby enabling a highly effective link between the research team and both NHS services and IT systems [63].

On completion of data collection and subsequent analyses, the findings from the trial will make a significant contribution to the evidence base for Counselling for Depression as contrasted with CBT, which is considered the front-line psychological intervention for depression. As such, the findings will be of importance to all stakeholders, whether they are commissioners purchasing services, practitioners delivering therapies, or patients seeking a choice and receiving such interventions.

### Trial Status

The study commenced recruitment in October 2014 and patient recruitment is on-going.

## Additional file

Additional file 1: SPIRIT 2013 Checklist: Recommended items to address in a clinical trial protocol and related documents. (DOC 123 kb)

## Abbreviations

BDI-II: Beck Depression Inventory II
CACE: Complier-adjusted causal effect
CBT: Cognitive behaviour therapy
CD-RISC: Connor-Davidson Resilience Scale
CfD: Counselling for depression
CIS-R: Clinical Interview Schedule-Revised
CORE-OM: Clinical Outcomes in Routine Evaluation-Outcome Measure
CSQ: Client Satisfaction Questionnaire
CSRI: Client Service Receipt Inventory
CTS-R: Cognitive Therapy Scale-Revised
EQ-5D-5L: Euroqol 5D-5L
GAD-7: Generalised Anxiety Disorder-7
IAPT: Improving Access to Psychological Therapies
ITT: Intention to treat
MINI: Mini International Neuropsychiatric Interview
NHS: National Health Service
PCEPS: Person-Centred and Experiential Psychotherapy Scale
PHQ-9: Patient Health Questionnaire-9
PP: Per-protocol
PPI: Public and patient involvement
PRaCTICED: Pragmatic Randomised Controlled Trial assessing the non-Inferiority of Counselling and its Effectiveness for Depression
SACS-CBT: Session Adherence and Competence Scale-CBT
SACS-CfD: Session Adherence and Competence Scale-CfD
WSAS: Work and Social Adjustment Scale
